# Effects of hearing impairment and hearing aid use on the incidence of cognitive impairment among community-dwelling older adults: evidence from the Taiwan Longitudinal Study on Aging (TLSA)

**DOI:** 10.1186/s12877-021-02012-4

**Published:** 2021-01-22

**Authors:** Chi-Jung Tai, Tzyy-Guey Tseng, Yu-Han Hsiao, Tsu-Ann Kuo, Ching-Ya Huang, Yi-Hsin Yang, Meng-Chih Lee

**Affiliations:** 1 Department of Family Medicine, Pingtung Hospital, Ministry of Health and Welfare, Pingtung, Taiwan; 2 Graduate Institute of Natural Products, College of Pharmacy, Kaohsiung Medical University, Kaohsiung, Taiwan; 3 Department of Family Medicine, Kaohsiung Medical University Hospital, Kaohsiung Medical University, Kaohsiung, Taiwan; 4 Department of Family Medicine, Taichung Hospital, Ministry of Health and Welfare, 199, sec. 1, San-Min Road, Taichung, Taiwan; 5 Department of Public Health, Chung Shan Medical University, Taichung, Taiwan; 6 College of Management, Chaoyang University of Technology, Taichung, Taiwan; 7 Department of Medical Sociology and Social Work, Chung Shan Medical University, Taichung, Taiwan; 8 School of Pharmacy, College of Pharmacy, Kaohsiung Medical University, Kaohsiung, Taiwan; 9 National Institute of Cancer Research, National Health Research Institutes, Tainan, Taiwan; 10 Institute of Population Health Sciences, National Health Research Institutes, Miaoli, Taiwan

**Keywords:** Cognitive impairment, Frailty, Geriatric syndromes, Hearing aid, Hearing impairment

## Abstract

**Background:**

Previous studies have reported associations between hearing impairment (HI) and cognitive impairment, but the evidence is not conclusive while considering concurrent geriatric syndromes. Especially, evidence from previous studies rarely came from Asian studies. This study aimed to evaluate the independent effects of HI and hearing aid use on the incidence of cognitive impairment while considering most geriatric confounders.

**Methods:**

This population-based, propensity-score matched cohort study used cohort from Waves IV–VII (1999–2011) survey of the Taiwan Longitudinal Study on Aging (TLSA). Cognitive impairment was identified based on Short Portable Mental Status Questionnaire (SPMSQ) scores. The hazard ratio (HR) was calculated using the Cox proportional hazard regression adjusting for age, sex, comorbidities, socioeconomic status, Center for Epidemiologic Studies Depression (CES-D) scores, the instrumental activities of daily living scale, mobility condition and quality of life. In addition, social support and participation were also considered as confounders in the analysis. To assess the robustness of our findings, we conducted a sensitivity analysis designed to access unmeasured confounding factors by calculating E-values.

**Results:**

After 1:1 propensity-score matching, we included 709 participants in both the HI and non-HI groups with a mean age of 73.4 years and 39.4% of participants were female. The mean follow-up was 8.9 ± 3.9 years. The HI group had a higher incidence of cognitive impairment than the non-HI group (74.5% vs. 69.1%, respectively), with an adjusted HR of 1.16 (95% confidence interval [CI], 1.03–1.32) based on a 12-year follow up. The E-value was 1.45 for the estimate, which provided evidence for this study’s robustness. Although, a subgroup analysis showed that hearing aid use was associated with lower incidences of cognitive impairment (66.3% vs. 75.6%) when compared to non-users in the HI group, the adjusted HR of 0.82 (95% CI, 0.61–1.09) revealed no significant differences.

**Conclusions:**

HI was an independent risk factor of incident cognitive impairment on top of concurrent geriatric syndromes. Early HI detection may thus be effective for preventing cognitive decline. Further studies are needed to evaluate the effect of hearing aid use on the prevention of cognitive decline.

## Background

As it poses declines in memory and other cognitive functions, dementia creates high social and economic burdens for the ageing and those living in aged societies [1]. Many clinicians have therefore tried to identify modifiable risk factors for use in early interventions aimed at reducing the incidence of cognitive impairment. In this context, a variety of recent studies have focused on the association between age-related hearing impairment (HI) and cognitive impairment or dementia. This is because HI affects communication and can contribute to isolation, depression, and, possibly, dementia. Additionally, some types of HI is reversible with rehabilitative treatments such as hearing aid use and cochlear implantation [2].

In the United States, Lin and colleagues reported that HI was associated with 24% increased risk for incident cognitive impairment [3]. Deal and colleagues reported that moderate/severe HI was associated with increased risk of dementia (hazard ratio [HR], 1.55; 95% confidence interval [CI], 1.10–2.19) among older adults [4]. Similarly, Gurgel and colleagues demonstrated that HI was an independent predictor for developing dementia (HR, 1.27; 95%CI, 1.03–1.56) [5]. Further, the English Longitudinal Study of Ageing (ELSA) revealed that participants with self-reported HI had an odds ratio (OR) of 1.6 times (95%CI, 1.1–2.4) those with normal hearing in regard to developing dementia [6]. In Korea, Kim and colleagues found that both severe (adjusted HR, 1.17) and profound (adjusted HR, 1.51; 95% CI, 1.14–2.00) HI was associated with elevated dementia risks for middle- and older-aged individuals [7]. Heywood and colleagues used Singapore Longitudinal Ageing Study (SLAS) to show that HI was associated with increased prevalence of dementia (OR, 3.65; 95% CI, 1.16–11.4), and with high risk of developing mild cognitive impairment or dementia (HR, 2.30; 95% CI, 1.08–4.92) [8].

It is well-known that older adults often suffer from multi-morbidities. A study showed that among 2618 participants, 75.3, 41.8, and 22.5% had geriatric syndromes, multimorbidity, and disability, respectively, and 10.4% had all the three conditions” [9]. Recently, researchers have tried to use model adjustment or mediation analyses to assess possible mediating pathways between HI and cognitive impairment. For example, Fischer and colleagues attempted to prove that HI was independently associated with cognitive impairment (HR, 1.90; 95% CI, 1.11–3.26) while adjusting vision and olfaction impairments and frailty scores. However, there is residual confounding in the exposure-outcome relationship between HI and cognitive impairment as well-known confounders such as depression, physical function, and social support were not included in the analysis [10].

The results of several previous studies also conflict in regard to the effects of hearing aid interventions on cognitive function. For instances, Dawes and colleagues reported that hearing aid use was associated with better cognition independently of social isolation and depression [11]. Similarly, a population-based longitudinal cohort study showed that hearing aid use was positively associated with episodic memory scores [12]. On the other hand, Lin and colleagues indicated that self-reported hearing aid use was not associated with higher cognitive test scores among participants with hearing loss [13]. Moreover, another study found no significant differences between hearing-aid users and non-users in regard to cognitive issues, social engagement, or mental health outcomes among community-dwelling older adults with HI [14].

As such, this study aimed to evaluate the independent effects of HI and hearing aid use on the incidence of cognitive impairment among community-dwelling older adults while considering most geriatric confounders.

## Methods

### Study population

This study’s population included participants of the TLSA, which was a longitudinal, population-based survey initiated by the Health Promotion Administration, Ministry of Health and Welfare, Taiwan [15]. A three-stage systematic random sampling design was used to select an equal probability adult samples aged 60 or above. Data were collected through face-to-face personal interviews conducted by trained interviewers. Respondent follow-ups were then conducted every 3 to 4 years. A total of seven surveys were conducted in 1989, 1993, 1996, 1999, 2003, 2007, and 2011 (i.e., Waves I–VII). The details and design of the TLSA have been described elsewhere [16]. This study analyzed datasets from Waves IV–VII (i.e., 1999–2011).

**Fig. 1:**
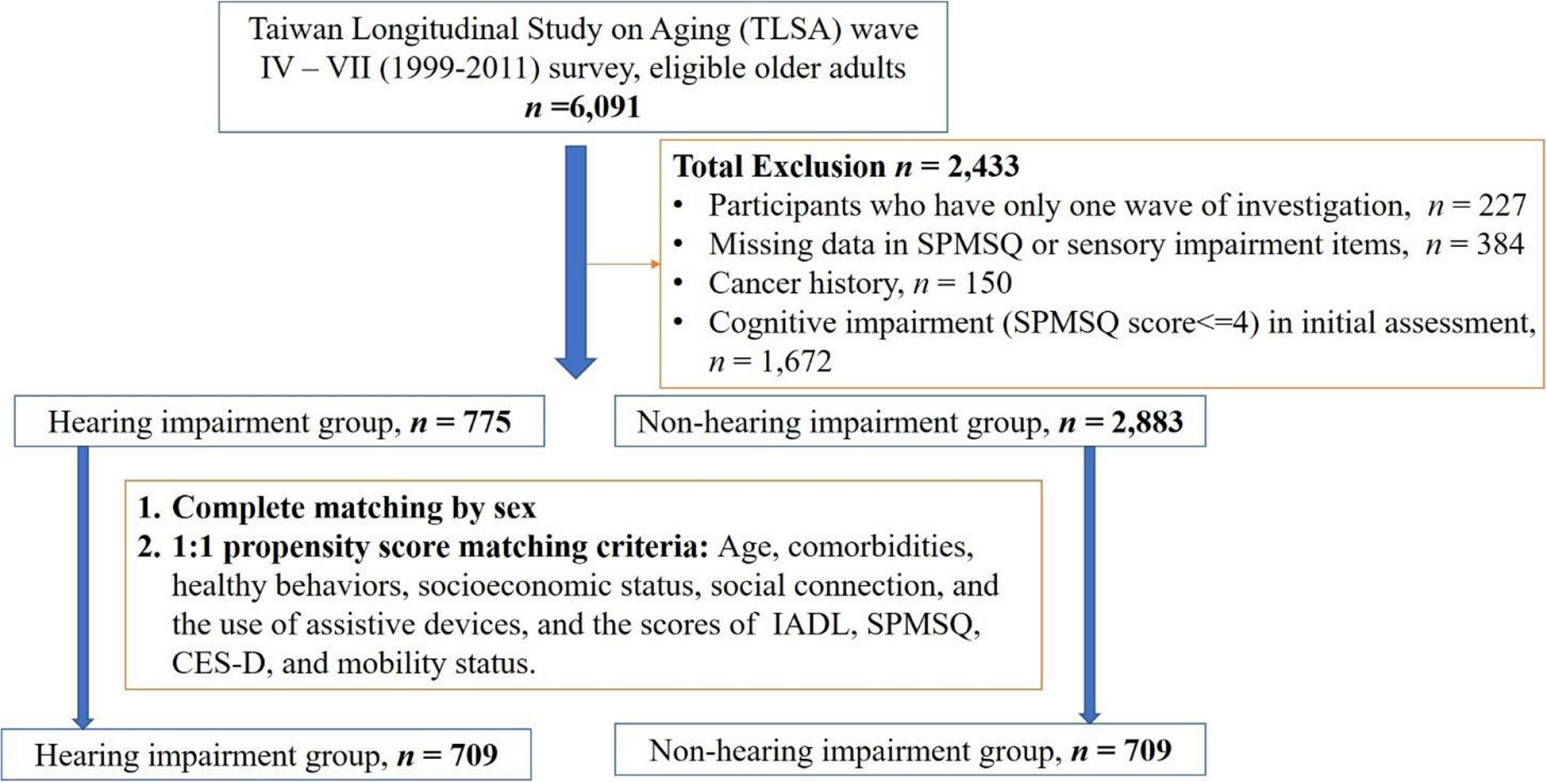
Flow chart showing the patient inclusion and exclusion processes and propensity-score matching criteria. CES-D, Center for Epidemiologic Studies Depression; IADL, instrumental activities of daily living scale; SPMSQ, short portable mental status questionnaire

First, of the 6091 eligible older adults, we excluded 227 individuals who had only completed one wave of investigation (Fig. 1). Second, we excluded 384 individuals due to missing data for either the Short Portable Mental Status Questionnaire (SPMSQ) or sensory impairment items. Third, we excluded 156 individuals with a history of cancer. Finally, we excluded 1672 individuals with cognitive impairments as defined by receiving SPMSQ scores ≤4 during their initial assessments [17]. Exclusion of older adults with cognitive impairments could ensure the accuracy of the responses in the initial assessments which may be influenced by cognitive status. Ultimately, data from 3658 older adults without cognitive impairments were included for analysis. HI was defined by self-reported hearing loss or hearing aid use. The final sample was then divided into HI (*n* = 775) and non-HI (*n* = 2883) groups.

### Dependent and independent variables

We gathered subject data on age, sex, height, weight, level of education, marital status, living arrangement, self-rated health condition, health-related behaviors, self-reported economic pressure, concurrent comorbidities, and the use of assistive device (Table 1).

**Table 1:** Baseline participant characteristics before propensity-score matching

	HI group *n* = 775	Non-HI group *n* = 2883	*P-*Value
Age^a^, year	74.3 ± 8.1	66.2 ± 9.3	< 0.001
Female sex, n (%)	300 (38.7%)	1317 (45.7%)	< 0.001
Height^a^, cm	159.7 ± 8.8	160.1 ± 8.1	0.20
Weight^a^, kg	59.2 ± 10.7	61.3 ± 10.7	< 0.001
Body mass index^a^, kg/m^2^	23.2 ± 3.4	23.9 ± 3.4	< 0.001
Assistive devices, n (%)			
Eyeglasses	388 (50.1%)	1809 (62.8%)	< 0.001
Hearing aids	94 (12.1%)	NA	
Dental prostheses	584 (75.4%)	2078 (72.1%)	0.07
Crutches	131 (16.9%)	208 (7.2%)	< 0.001
Living arrangements, n (%)			< 0.001
Home	758 (97.8%)	2862 (99.3%)	
Nursing home	17 (2.2%)	21 (0.7%)	
Educational level, n (%)			< 0.001
Elementary school or below	634 (81.8%)	1903 (66.0%)	
Junior high school	62 (8.0%)	346 (12.0%)	
Senior high school	64 (8.3%)	464 (16.1%)	
College or above	15 (1.9%)	170 (5.9%)	
Marital status, n (%)			< 0.001
Married/Cohabitating	456 (58.8%)	2081 (72.2%)	
Single/Divorced/Widowed	319 (41.2%)	802 (27.8%)	
Living conditions, n (%)			0.12
Living alone	86 (11.1%)	267 (9.3%)	
Living with spouse or children	689 (88.9%)	2616 (90.7%)	
Self-rated health condition, n (%)			< 0.001
Good or very good	158 (20.4%)	1223 (42.4%)	
Okay	300 (38.7%)	1022 (35.5%)	
Bad or very bad	317 (40.9%)	638 (22.1%)	
Self-reported economic pressure, n (%)			0.18
No	527 (68.0%)	1987 (68.9%)	
Some	158 (20.4%)	624 (21.6%)	
Heavy	90 (11.6%)	272 (9.4%)	
Health-related behaviors, n (%)			
Smoking	215 (27.7%)	669 (23.2%)	0.009
Alcohol	196 (25.3%)	894 (31.0%)	0.22
Betel nuts	54 (7.0%)	154 (5.3%)	0.08
Exercise habits, n (%)			0.004
No	288 (37.2%)	897 (31.1%)	
Exercise < 3 time/week	57 (7.4%)	265 (9.2%)	
Exercise ≥3 times/week	430 (55.5%)	1721 (59.7%)	
Comorbidities, n (%)			
Hypertension	309 (39.9%)	900 (31.2%)	< 0.001
Diabetes mellitus	113 (14.6%)	385 (13.4%)	0.38
Heart disease	171 (22.1%)	434 (15.1%)	< 0.001
Stroke	52 (6.7%)	86 (3.0%)	< 0.001
COPD	143 (18.5%)	258 (9.0%)	< 0.001
Arthritis	183 (23.6%)	468 (16.2%)	< 0.001
Gastric ulcer	169 (21.8%)	503 (17.5%)	0.005
Hepatobiliary disease	64 (8.3%)	220 (7.6%)	0.56
Hip fracture	23 (3.0%)	41 (1.4%)	0.004
Cataract	219 (28.3%)	608 (21.1%)	< 0.001
Chronic kidney disease	69 (8.9%)	230 (8.0%)	0.40
Spine spondylosis	71 (9.2%)	271 (9.4%)	0.84
Physical and psychosocial status^a^			
Social support score	15.7 ± 3.2	16.4 ± 3.0	< 0.001
Social participation	0.42 ± 0.49	0.45 ± 0.50	0.14
Mobility score	2.6 ± 2.5	1.2 ± 1.9	< 0.001
IADL score	0.75 ± 1.32	0.26 ± 0.85	< 0.001
CES-D score	4.5 ± 5.3	2.8 ± 4.3	< 0.001
SPMSQ score	8.3 ± 1.1	8.5 ± 0.9	< 0.001
Quality of Life score	7.5 ± 3.1	8.3 ± 2.9	< 0.001

Higher total scores on the social support scale (0–20), social participation scale (0–6), SPMSQ (0–9), and Quality of Life scale (0–12) represented better condition. On the other hand, lower total scores on the mobility scale (0–8), IADL scale (0–5), and CES-D (0–24) represented better condition. Details are available in Methods section of the article

*CES-D* Center for Epidemiologic Studies Depression, *COPD* Chronic obstructive pulmonary disease, *HI* Hearing impairment, *IADL* Instrumental activities of daily living scale, *SPMSQ* Short portable mental status questionnaire

^a^ Values are given as means ± standard deviations, not no. (%)

This study evaluated respondent physical and psychosocial status using multidimensional scores from the social support scale, social participation scale, mobility scores, instrumental activities of daily living (IADL) scale, Center for Epidemiologic Studies Depression (CES-D) scale, SPMSQ, and quality of life (QoL) scale [18–22]. Higher total scores for the social support scale (0–20), social participation scale (0–6), SPMSQ (0–9), and QoL scale (0–12) represented better condition. On the other hand, lower total scores for mobility scale (0–8), IADL scale (0–5), and CES-D (0–24) represented better condition. The details of each scales are given below.

The social support scale consisted of four questions designed to measure the four corresponding items of emotional support (What degree of care is received from family members, relatives, and/or friends?), instrumental support (Is someone available if you need help?), and information support (Do you feel that your family provides useful help? and Does your family consult your opinion when they make decisions?) [22]. Each item was scored from 1 to 5, with total scores ranging from 0 to 20.

Respondents answered the social participation scale to indicate whether they participated in group activities through one or more of six types of social organizations (i.e., hobby related clubs, religious or church groups, political groups, retired associations, elderly-related associations, and volunteer groups) [19]. Each type was coded as yes (score = 1) or no (score = 0), with total scores ranging from 0 to 6.

For physical function assessments, the mobility scale included the difficulty level of standing for 15 min, stooping, reaching overhead, grasping with fingers, lifting or carrying 25 pounds, running for 20–30 m, walking 200–300 m, and climbing stairs [20]. The IADL scale was used to assess five activities as proposed by Lawton and Brody [21]. Respondents who reported difficulty, were unable to carry out tasks, or received help and/or used equipment when performing tasks were coded as having difficulty (i.e., 1 = yes, 0 = no).

Depressive symptoms were measured using the shorter form of the CES-D scale [23]. This included the eight items of “my appetite was poor,” “everything I did took effort,” “my sleep was restless,” “felt depressed,” “felt lonely,” “people were unfriendly,” “felt sad,” and “could not get going.” Responses ranged from “rarely or none of the time” to “most or all of the time” to produce scores between 0 and 3, respectively, with total scores ranging from 0 to 24 [20].

The QoL scale consisted of 12 items proposed by Neugarten and colleagues [24], including “is your life better than that of most others?,” “are you satisfied with your life?,” “are you interested in the things in which you are engaged?,” “have the most recent years contained the best days of your life?,” “would you like to change your past life?,” “do you expect something good to happen in the future?,” “should your life be better than it is now?,” “are you bored with most things you do?,” “do you feel old and tired?,” “does most of your life meet your expectations?,” “do you feel that you are living in a safe environment?,” and “are you satisfied with your living environment?” Each item was rated as yes (1) or no (0), with total scores ranging from 0 to 12.

The dependent variable of this study was the incident occurrence of a > 4 point deficit on the SPMSQ, while the independent variable was self-reported hearing deficit. This study applied the modified version of SPMSQ to the exclusion criteria, propensity-score matching covariates, and primary outcome to constitute criteria for cognitive impairment. This study included responses to the nine following SPMSQ items: “where are you located now?,” “what is your home address?,” “what day is it?,” “what month is it?,” “what year is it?,” “how old are you?,” “who are the current and last presidents?,” and “subtract 3 from 20 four consecutive times” [17]. Participants with four or more errors were described as having cognitive impairment, which was our primary outcome, and several other cohort studies supported this cut-off point [25].

### Sensitivity analysis

We assessed the robustness of our findings by conducting a sensitivity analysis designed to access unmeasured confounding factors related to the exposures and outcomes by calculating E-values [26]. The E-value is the minimum strength of association, on the risk ratio scale, that unmeasured confounders would need to have with both the exposure and outcome, conditional on the measured covariates, to fully explain away a specific exposure-outcome association.

### Statistical analyses

Propensity-score matching was conducted through the PSMATCH procedure provided by SAS (Statistics Analysis System Institute Inc., Cary, NC, USA). This study used descriptive statistics to assess patient demographics. The adjusted HRs of incidences of cognitive impairment were calculated using the Cox proportional hazard model while adjusting for possible confounders. All analyses were conducted using SAS version 9.4.

## Results

Prior to the propensity-score matching process, the HI group contained 775 respondents, while the non-HI group contained 2883 respondents (Fig. 1). HI respondents were significantly older (74.3 vs. 66.2 years). Further, the HI group was associated with lower self-rated health, less regular exercise, higher smoking rates, and higher prevalence rates for hypertension, heart disease, stroke, arthritis, gastric ulcers, hip fractures, and cataracts (Table 1). Most importantly, HI respondents generally had poorer physical and psychosocial status based on evaluations of social support, mobility, IADL, CES-D, SPMSQ, and QoL (Table 1).

After matching with the covariates, we included 709 participants in both the HI and non-HI groups for further analysis. All covariates were well-balanced between groups (Table 2). The mean follow-up was 8.9 ± 3.9 years. In the HI and non-HI groups, 228 (32.2%) vs. 211 (29.8%), 156 (22.0%) vs. 145 (20.5%), and 325 (45.8%) vs. 353 (49.8%) patients had 4-year, 8-year, and 12-year follow-ups, respectively. In total, the HI and non-HI groups contributed 6060 and 6240 person-years of follow-up. Respondent results for the mobility, IADL, CES-D, SPMSQ, and QoL scales showed that most had good physical and psychological function (Table 2).

**Table 2:** Baseline characteristics of participants after 1:1 propensity-score matching

	HI group *n* = 709	Non-HI group *n* = 709
Age^a^, year	73.4 ± 7.7	73.5 ± 7.7
Female sex, n (%)	279 (39.4%)	279 (39.4%)
Height^a^, cm	159.7 ± 8.8	159.7 ± 8.4
Weight^a^, kg	59.4 ± 10.7	59.8 ± 10.4
Body mass index^a^, kg/m^2^	23.2 ± 3.3	23.4 ± 3.6
Assistive devices, n (%)		
Eyeglasses	366 (51.6%)	377 (53.2%)
Hearing aids	83 (11.7%)	NA
Dental prostheses	534 (75.3%)	536 (75.6%)
Crutches	109 (15.4%)	114 (16.1%)
Living arrangements, n (%)		
Home	694 (97.9%)	693 (97.7%)
Nursing home	15 (2.1%)	16 (2.3%)
Educational level, n (%)		
Elementary school or below	570 (80.4%)	557 (78.6%)
Junior high school	61 (8.6%)	66 (9.3%)
Senior high school	64 (9.0%)	66 (9.3%)
College or above	14 (2.0%)	20 (2.8%)
Marital status, n (%)		
Married/Cohabitating	431 (60.8%)	424 (59.8%)
Single/Divorced/Widowed	278 (39.2%)	285 (40.2%)
Living conditions, n (%)		
Living alone	82 (11.6%)	82 (11.6%)
Living with spouse or children	627 (88.4%)	627 (88.4%)
Self-rated health condition, n (%)		
Good or very good	156 (22.0%)	166 (23.4%)
Okay	280 (39.5%)	266 (37.5%)
Bad or very bad	273 (38.5%)	277 (39.1%)
Self-reported economic pressure, n (%)		
No	482 (68.0%)	483 (68.1%)
Some	144 (20.3%)	150 (21.2%)
Heavy	83 (11.7%)	76 (10.7%)
Health-related behaviors, n (%)		
Smoking	195 (27.5%)	186 (26.2%)
Alcohol	185 (26.1%)	176 (24.8%)
Betel nuts	44 (6.2%)	46 (6.5%)
Exercise habits, n (%)		
No	260 (36.7%)	254 (35.8%)
Exercise < 3 time/week	48 (6.8%)	36 (5.1%)
Exercise ≥3 times/week	401 (56.6%)	419 (59.1%)
Comorbidities, n (%)		
Hypertension	273 (38.5%)	279 (39.4%)
Diabetes mellitus	105 (14.8%)	116 (16.4%)
Heart disease	155 (21.9%)	156 (22.0%)
Stroke	44 (6.2%)	45 (6.4%)
COPD	117 (16.5%)	122 (17.2%)
Arthritis	161 (22.7%)	155 (21.9%)
Gastric ulcer	152 (21.4%)	150 (21.2%)
Hepatobiliary disease	56 (7.9%)	61 (8.6%)
Hip fracture	19 (2.7%)	17 (2.4%)
Cataract	199 (28.1%)	204 (28.8%)
Chronic kidney disease	66 (9.3%)	65 (9.2%)
Spine spondylosis	67 (9.5%)	68 (9.6%)
Physical and psychosocial status^a^		
Social support score	15.8 ± 3.2	15.8 ± 3.4
Social participation	0.42 ± 0.49	0.45 ± 0.50
Mobility score	2.4 ± 2.5	2.4 ± 2.4
IADL score	0.64 ± 1.23	0.65 ± 1.31
CESD score	4.3 ± 5.2	4.1 ± 5.1
SPMSQ score	8.3 ± 1.1	8.3 ± 1.2
Quality of Life score	7.5 ± 3.0	7.5 ± 3.1

Higher total scores on the social support scale (0–20), social participation scale (0–6), SPMSQ (0–9), and Quality of Life scale (0–12) represented better condition. On the other hand, lower total scores on the mobility scale (0–8), IADL scale (0–5), and CES-D (0–24) represented better condition. Details are available in Methods section of the article

*CES-D* Center for Epidemiologic Studies Depression, *COPD* Chronic obstructive pulmonary disease, *HI* Hearing impairment, *IADL* Instrumental activities of daily living scale, *SPMSQ* Short portable mental status questionnaire

^a^ Values are given as means ± standard deviations, not no. (%)

The incidence of cognitive impairment was statistically higher for the HI group (74.5% vs. 69.1%) based on the 12-year follow-up, with an adjusted HR of 1.16 (95% CI, 1.03–1.32) (Table 3). Further, there was no difference in the risk of cognitive impairment between groups based on the 4- (HR, 1.13;95% CI, 0.94–1.35) and 8-year (HR, 1.11; 95% CI, 0.97–1.28) follow-ups. A subgroup analysis revealed that HI respondents who used hearing aids had lower incidences of cognitive impairment (66.3% vs. 75.6%) when compared to those who did not use hearing aid during the 12-year follow-up (Table 4). However, the adjusted HR showed no significant decreases in hearing aid users when compared to non-users (Table 4).

**Table 3:** Comparison of cognitive impairment incidence between the hearing impairment and non-hearing impairment groups

Incidence of cognitive impairment	HI group *N* = 709	Non-HI group *N* = 709	Adjusted HR (95% CI)	*P* Value
4-year follow-up	259 (36.5%)	230 (32.4%)	1.13 (0.94–1.35)	.18
8-year follow-up	414 (58.4%)	383 (54.0%)	1.11 (0.97–1.28)	.14
12-year follow-up	528 (74.5%)	490 (69.1%)	1.16 (1.03–1.32)	.02^a^

Hazard ratio was adjusted for all covariates listed in Table 2

*CI* Confidence interval, *HI* Hearing impairment, *HR* Hazard ratio

^a^ Cox proportional hazard regression, *p* < 0.05

**Table 4:** Comparison of cognitive impairment incidence between hearing impairment participants (with and without hearing aids)

Incidence of cognitive impairment	Hearing aid (+) *N* = 83	Hearing aid (−) *N* = 626	Adjusted HR (95% CI)	*P* Value
4-year follow-up	27 (32.5%)	232 (37.1%)	0.93 (0.61–1.42)	.74
8-year follow-up	45 (54.2%)	369 (59.0%)	0.81 (0.58–1.11)	.19
12-year follow-up	55 (66.3%)	473 (75.6%)	0.82 (0.61–1.09)	.17

Hazard ratio was adjusted for all covariates listed in Table 2

*CI* Confidence interval, *HR* Hazard ratio

### Sensitivity analyses

We used the formula to calculate the E-value for the effect estimate [26, 27]. For unmeasured confounders associated with HI and cognitive impairment during the 12-year follow-up, the E-value formula produced *E* = 1.45 for the estimate. The result could be interpreted that an unmeasured confounder that was associated with both the HI and cognitive impairment by a risk ratio of 1.45-fold each, above and beyond the measured confounders, but weaker confounding would not do so. As such, the E-value provided evidence for this study’s robustness.

## Discussion

To the best of our knowledge, this was the first study to demonstrate that HI elevated the risk of cognitive impairment among community-dwelling older adults while controlling for most concurrent geriatric confounders. Over 12 years, community-dwelling older adults with HI had significant increased risks for the incidence of cognitive impairment when compared to those without HI. By contrast, older adults with HI did not show an increased risk of cognitive impairment based on the 4- and 8-year follow-ups. We supposed that was because the development of cognitive impairment took time, and HI was only one of the factors resulting in cognitive impairment. Therefore, the significant difference of incident cognitive development between the HI and non-HI group could not easily show in short follow-up period. This assumption was supported by previous cohort studies, which demonstrated that older adults with HI have significantly increased HR of 1.55 over 9 years [4], 1.39–1.59 over 11 years [6], and 1.27 over 12.5 years of follow-up [5]. This study produced similar results, but with a lower HR of 1.16, because we matched and adjusted for most geriatric physical and psychosocial confounders known to increase the risk of cognitive impairment.

Age-related HI is characterized by high-frequency impairment (6000 and 8000 Hz), which may originate in the peripheral or central auditory systems [28]. However, most early-stage HI goes undetected for older adults. Further, those with self-reported HI often suffer from notably decreased decibels in hearing level (dB HL). A previous study showed that individuals with HI require 7.7 years on average to develop cognitive impairments, vs 10.9 years for individuals with normal hearing [3]. This study also showed that older adults with HI had an increased risk of cognitive impairment when compared to older adults without HI based on 12 years of follow-up. This has substantial public health policy implications. That is, there is a sufficient time period in which to detect and manage HI among these individuals, thus potentially preventing incidences of cognitive impairment.

Clinical practice recommendations entail that hearing function should be tested during comprehensive geriatric assessments [29]. However, the importance of the Rinne tuning-fort test results are often underestimated in general practice when compared to cardiovascular risk factors. Moreover, only patients with significant HI (55 ~ 110 dBHL) are eligible for government subsidies to buy hearing aids. Notably, a previous study showed that moderate/severe HI (> 40 dBHL) was associated with incident dementia [4].

HI and cognitive decline may share age-related neurodegenerative mechanisms [30]. In addition, the ‘cascade hypothesis’ supposed that HI had impact on cognition in older adults either directly through impoverished auditory input, or via effects of HI on social isolation and depression [11, 31]. A systematic review also showed that communication breakdown resulted from HI was associated with loneliness and social isolation, which had important implications for the cognitive and psychosocial health of older adults [32]. Although the benefits of hearing aids in regard to preventing cognitive impairments are not yet evident, these devices may still prevent social isolation and depression [11].

An ongoing randomized study, Aging and Cognitive Health Evaluation in Elders (ACHIEVE) trial, aims to determine efficacy of a best practices hearing (vs. successful aging) intervention on reducing cognitive decline in older adults with hearing loss [33]. In addition, Hearing Aids to Support Cognitive Functions of Older Adults at Risk of Dementia (HearCog) trial has been designed to evaluate whether correction of hearing loss through the use of hearing aids decreases the 12-month rate of cognitive decline among older adults at risk of dementia [34]. Further trials are also needed to evaluate the cost-effectiveness of hearing aids in the dementia prevention and management contexts.

### Limitations

Although this study generated important findings, they should be interpreted with some cautions. First, although we used multiple strategies to minimize the effects of confounders through propensity-score matching, this observational study may entail residual confounding factors and thus cannot prove causality. Second, we did not evaluate HI severity and possible mechanisms of HI among respondents, which was related to cognitive impairment. In addition, the severity of HI may make progress through the follow-ups, which was likely to affect the SPMSQ score. However, it is more important to detect HI during community screening than it is to determine severity. Third, although two groups were balanced at the baseline, some time-varying changes including later onset of hearing loss in the non-HI group could influence the result. However, these effects could only make the HRs of our results underestimated. Therefore, the HI group may have higher risk of incident cognitive impairment. Fourth, compliance with hearing aids is often much less than with wearing glasses in the older adults [35]. Therefore, the effect of hearing aid on the prevention of cognitive decline might be underestimated. Finally, the sample size was relatively limited in regard to hearing aid users. Future clinical trials are therefore needed to confirm how hearing aid use may prevent cognitive decline.

## Conclusions

This study found that HI was associated with an increased risk for the incidence of cognitive impairment while controlling for most geriatric confounders based on 12 years of follow-up data. Therefore, integration of hearing test into annual health prevention program was an effective way to screen HI and prevent HI-associated cognitive decline among community-dwelling older adults.

## Data Availability

The datasets used and analyzed during the current study are not publicity available, but are available from the corresponding author on reasonable request with the permission of the Ministry of Health and Welfare, Taiwan.

## Abbreviations

CES-D: Center for Epidemiologic Studies Depression
CI: Confidence interval
dB HL: Decibels in hearing level
ELSA: English Longitudinal Study of Ageing
HR: Hazard ratio
HI: Hearing impairment
IADL: Instrumental activities of daily living
QoL: Quality of life
SPMSQ: Short Portable Mental Status Questionnaire
TLSA: Taiwan Longitudinal Study on Aging
